# Get In and Get Out: The Impact of COVID-19 on the Emotional Health of Home Care Workers

**DOI:** 10.1093/geroni/igab046.1475

**Published:** 2021-12-17

**Authors:** Leah Janssen

**Affiliations:** Scripps Gerontology Center, Oxford, Ohio, United States

## Abstract

This research explores the emotional health of home care workers (HCWs) during the coronavirus pandemic. In-depth, qualitative interviews were conducted with 17 home care workers in the fall of 2020. Thematic analysis revealed important connections between emotional health and success on the job. Perception of time and appreciation emerged as key elements that impacted emotional health. HCWs expressed the pressure to perform as usual while simultaneously taking on extra tasks as distracting from direct client care and reinforcing a task-oriented care approach. As a result of these tensions, HCWs experienced a loss of appreciation by the client, who prioritized personal safety and a “get in and get out” attitude, leaving the HCW feeling less fulfilled in their work. Implications of this research highlight the importance of HCW emotional health needs when retaining HCWs as valuable members of the long-term care workforce is paramount.

